# The effect of dexmedetomidine on inflammatory response of septic rats

**DOI:** 10.1186/s12871-015-0042-8

**Published:** 2015-05-01

**Authors:** Jianxing Zhang, Zhipeng Wang, Yan Wang, Guobin Zhou, Hongying Li

**Affiliations:** Guangdong General Hospital, Guangdong Academy of Medical Sciences, 510080 Guangzhou, Guangdong China

**Keywords:** Dexmedetomidine, Yohimbine, Sepsis, CLP, Inflammatory mediators

## Abstract

**Background:**

Some studies have demonstrated dexmedetomidine has anti-inflammatory effect on septic rats. However, the mechanism of how dexmedetomidine exerts these effects is still remained unknown. This study was designed to investigate the mechanism of how dexmedetomidine inhibits the production of inflammatory mediators in cecal ligation and puncturinduced septic rats.

**Methods:**

48 Sprague-Dawley rats were randomly divided into six groups: sham-operated (sham) group, cecal ligation and puncture (CLP) group, dexmedetomidine 5 μg/kg (DEX5) group, dexmedetomidine 10 μg/kg (DEX10) group,dexmedetomidine + yohimbine (DEX10 + Yoh) group and yohimibine group (Yoh). Blood, bronchoalveolarlavage fluid (BALF) and lung tissues in each group were collected at six hours after dexmedetomidine or yohimbine treatment,. Tumor necrosis factor-α (TNF-α) and interleukin-6 (IL-6) in BALF and plasma were measured by enzyme-linked immunosorbent assay (ELISA). Toll-like receptor-4(TLR4) and myeloid differerntiation factor(MyD88) expression were measuredby quantitative PCR, and extracellular signal-regulated kinase (ERK) 1/2 phosphorylation were determined by western blott.

**Results:**

Compared with CLP group, dexmedetomidine significantly decreased not only the production of TNF-α and IL-6 both in plasma and BALF, but also inhibited the expression of TLR4 and MyD88 in mRNA level and the activation of ERK1/2 and NF-κB in the lung tissues of CLP-induced septic rats. All these effects could not be reversed by yohimibine.

**Conclusions:**

Dexmedetomidine treatment can effectively reduce the generation of inflammatory mediators in the plasma and BALF of CLP-induced septic rats. These effects of dexmedetomidine rely on TLR4/MyD88/MAPK/ NF-κB signaling pathway and are independent of α_2_-adrenoceptor.

## Background

Sepsis is one of the major cases in intensive care unit(ICU) and has a very high mortality rate associated with hemodynamic instability, multiple organ dysfunctions (MODS), abnormal platelet and disseminated intravascular coagulation (DIC) [1]. All these pathophysiological process involves the uncontrolled over expression of inflammatory cytokines such as TNF-α,IL-8 and IL-6 et al. [2-5].

Dexmedetomidine is a selective α_2_-adrenergic receptor agonist, with sedative, antianxiety, sympatholytic and hemodynamic stability characteristics [6]. Somestudies have demonstrated that dexmedetomidine can reduce the dosage of other anesthesia-related drugs used in perioperative period and help to keep hemodynamic stable and almost has no respiratory depression effect during sedation or anesthesia [7-9]. In addition, dexmedetomidine has also been revealed to have anti-inflammation effect on septic rats [10]. However, the molecular mechanism of how dexmedetomidine exerts these effects in endotoxemia or sepsis is still remained unknown.

As the major receptors for patheogen-associated molecular patterens(PAMPs), Toll-like receptors(TLRs) belong to transmenbrane protein family and play a critical role in the regulation of inflammatory and innate immune responses [11]. Of them TLR4 is required for the recognition and subsequent signal transduction of LPS signal pathway [12,13]. After binding to TLR4, LPS activates multiple intracellular signaling molecules, including the mitogen-activated protein kinase (MAPK) family [extracellular signal-regulate kinase (ERK),c-Jun N-terminal kinase (JNK) or p38],and nuclear factor-κB (NF-κB) [14]. The activation of NF-κB induces the transcription of proinflammatory cytokines such as TNF-α and IL-6 [15].

In this study, we designed to investigate the effect of dexmedetomidine on TNF-α and IL-6production in the plasma and BALF of CLP induced septic rats. Since TLR4/MyD88/MAPK/NF-κB signal pathway plays a critical role in regulating the generation of inflammatory cytokines in endotoxemia or sepsis [16]. We also examed the effects of dexmedetomidine on these signal molecules in septic rats. We just hypothesized that the anti-inflammatory effect of dexmedetomidine is relevant to TLR4/MyD88/MAPK/NF-κB signal transduction pathway.

## Methods

### Animals

Male Sprague-Dawley rats (180-220 g) were obtained from the Southern Medical University Animal Center (Guangzhou,China). All animal procedures were approved and conducted in accordance with the guidelines for the care and use of animals of the ethics committee of Southern Medical University.

### Cecal Ligation and Puncture (CLP) operation

Rats were anesthetized by an intraperitoneal injection of pentobarbital sodium 50 mg/kg. Polymicrobial sepsis was induced by CLP as previously described [17]. The whole process was performed under sterile conditions with the abdominal skin sterilizedby75% alcohol. Laparotomy was conducted through 2 cm lower-midline incision. Cecum was exposed and ligated immediately distal to the ileocecal valve to avoid intestinal obstruction and then punctured twice with a 22-gauge needle, squeezed gently to force out a small amount of feces, and returned to the abdominal cavity. The abdomen is closed with 4-0 silk sutures in two layers.

### Experimental protocal and surgical procedures

Forty-eight Sprague-Dawley (SD) ratswere randomly divided into six groups: sham operation(sham group, n = 8), CLP (CLP group, n = 8), Dexmedetomidine 5 μg/kg(DEX5 group, n = 8), Dexmedetomidine 10 μg/kg(DEX10 group, n = 8), Dexmedetomidine 10 μg/kg + yohimbine1.0 mg/kg(DEX10 + Yoh group, n = 8), and yohimbine1.0 mg/kg(Yoh group, n = 8). Dexmedetomidine and yohimbine were injected through caudal vein immediately after the CLP or sham operation was done.

### Blood sample, bronchoalveolar Lavage (BAL) fluid and lung tissues collection

Six hours later, blood samples of the animals in each group were collected from inferior vena cava. The left lungs were removed and rinsed several times in ice-saline, then dried with filter paper, and stored at -80°C for subsequent protein detection. The right lungs were lavaged five times with 10 ml cold sterile saline through a tracheostomy tube. Then the BAL fluid(BALF) was collected and centrifuged at 3200 rpm, 4°C(Eppendorf 5804R; Eppendorf, Hamburg, Germany) to collected the supernatant for the measurement of TNF-α and IL-6by enzyme-linked immunosorbent assays (ELISA) according to the instruction of the kits(R&D Systems Inc, Minneapolis, MN, USA).

### Quatitative PCR(qPCR) assay of TLR4 mRNA and MyD88mRNA in lung tissuses

The mRNA expression of TLR4 and MyD88 in lung tissues was analyzed by qPCR. The total RNA was isolated from lung tissuses of each group by Trizol regent (Gibco-BRL), according to the manufactures’s instructions. 5 μg of total RNA was reverse-transcribed into cDNA, and the PCR reaction mixtures were prepared by SYBR Green qPCR master mix (Toyoho Co., Ltd., Osaka, Japan). β-actin was used as the internal control. Primers (Daan Gene, Guangzhou, China) were designed with sequences as follows: Forward: 5′- AACCCTAAGGCCAACAGTGAAAAG-3′ and reverse: 5′-TCATGAGGTAGTCTGTCAGGT-3′ for β-actin; and Froward: 5′-GCCGGAAAGTTATTGTGGTGGT-3′ and reverse: 5′-ATGGGTTTTAGGCGCAGAGTTT-3′for TLR4; and Forward: 5′-ACCGCATCGAGGAGGACTG-3′ and reverse: 5′-CTGTGGGACACTGCTCTCCA-3′ for MyD88. The relative expression levels of TLR4 and MyD88 mRNA in the lung tissues were determined by the2-ΔΔCT method.

### Western blot for P-ERK and NF-κB

Snap-frozen rats lung tissues(50 μg) was homogenized and centrifuged at 12,000 rpm at 4°C for 20 min, then the supernatant was collected. Nuclear extracts were collected to analyze the nuclear translocation of NF-κB p65 protein by western blot. The samples were normalized for equal amounts of total protein assayed by bicinchoninic acid(BCA) method. Fifty micrograms protein from each sample was separated on a sodium dodecyl sulfate-polyacrylamide gel and transferred to PVDF membranes. The membranes were blocked with 5% nonfat milk and incubated overnight with primary anti-Phospho-ERK antibody and anti-NF-κB p65 antibody at 4°C. Next day the membranes were incubated with the corresponding HRP-conjugated secondary antibody at room time for 1 hour. Cellular GAPDH protein was immunodetected as the internal standard.

### Statistical analysis

All data are presented as the mean ± standard deviation. The data was proved as normal distribution by Kolmogorov-Smimov test. (SPSS19. USA). Experimental results were analyzed by one-way analysis of variance with the Bonferroni test for variances. P < 0.05 was considered to indicate a statistically significant difference. Graphs and figures were madewith Graphpad Prism 5.01 (GraphPad software, CA, USA).

## Result

### Dexmedetomidine reduced the generation of TNF-α and IL-6 in the BALF and plasma

**Figure 1:**
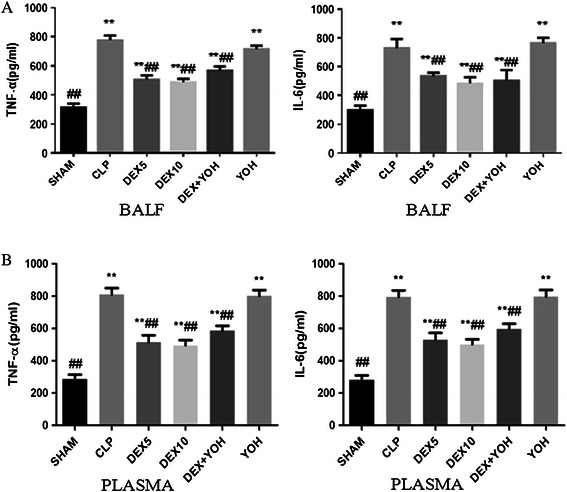
ELISA analysis of TNF-α and IL-6 expression in BALF and plasma. Rats were treated with dexmedetomidine (5 μg/kg or 10 μg/kg), or yohimbine(1.0 mg/kg) for six hours after CLP or operation. ELISA analysis of TNF-α, and IL-6 expression in BALF **(A)** and plasma **(B)**. The data were presented as the mean ± standard deviation.*P < 0.05 and **P < 0.01 vs. the sham group; ^#^P < 0.05 and ^##^P < 0.01vs. CLP group,n = 8.

As shown in Figure 1, TNF-α and IL-6 in the BALF and plasma of CLP and yohimibine groups were increased obviously, compared with those of the sham group (P < 0.05). Yohimibine had no effect on the production of TNF-α and IL-6 in the BALF and plasma of CLP-induced sepsis (P > 0.05). As we expected, dexmedetomidine (5 μg/kg and 10 μg/kg) effectively inhibited the increase of TNF-α and IL-6 in the plasma and BALF of CLP-induced septic rats (P < 0.01). This effect of dexmedetomidine could not be reversed by yohimibine (a kind of α_2_-adrenoceptor antagonist). All these results indicated that dexmedetomidine can inhibit the generation of inflammatory mediators such as TNF-α and IL-6 during sepsis and this effect is independent of its α_2_-adrenoceptor activation effect.

### Dexmedetomidine inhibited the expression of TLR4 and MyD88 in the lung tissues of CLP-induced septic rats

**Figure 2:**
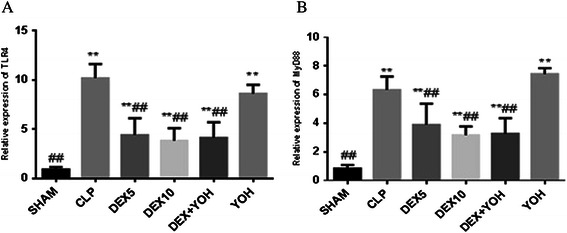
The expression of TLR4 mRNA **(A)** and Myd88 mRNA **(B)** in lung tissues. Rats were treated with dexmedetomidine (5 μg/kg or 10 μg/kg), or yohimbine(1.0 mg/kg) for six hours after CLP or operation. The expression of TLR4 mRNA **(A)** and Myd88 mRNA **(B)** in lung tissues were measured by RT-PCR . Datas were presented as the mean ± standard deviation. *P < 0.05 and **P < 0.01 vs. the sham group; ^#^P < 0.05 and ^##^P < 0.01vs. CLP group, n = 8.

Since TLR4 is required for LPS recognition and MyD88 is important for the downstream signal transduction, we measured the expression of TLR4 and MyD88 in lung tissues of all the six groups. Compared with sham group, CLP-induced sepsis dramatically increased TLR4 and MyD88 mRNA expression in both CLP and yohimibine groups (P < 0.01). Meanwhile dexmedetomidine with a dose of either 5 μg/kg or 10 μg/kg significantly inhibited CLP-induced TLR4 and MyD88 mRNA expression and this effect could not be reversed by yohimbine (P < 0.01) (Figure 2).

### Dexmedetomidine inhibited the activation of ERK1/2 and NF-κB

**Figure 3:**
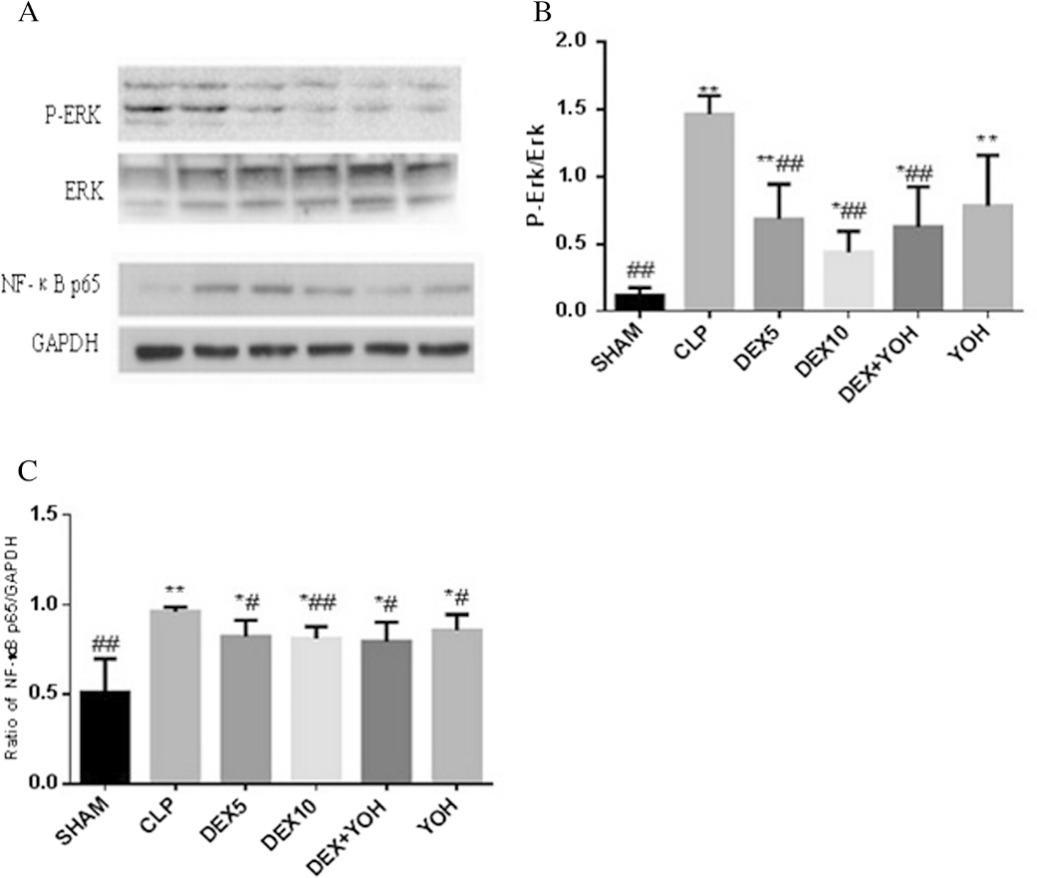
Western blot analysis of P-ERK and NF-κB p65. Rats were treated with dexmedetomidine (5 μg/kg or 10 μg/kg), or yohimbine(1.0 mg/kg) for six hours after CLP or operation. P-ERK and NF-κB p65 activation in lung tissues were analyzed by Western blot **(A)**; The correspondingly gray intensity analysis of the western blot were shown in **(B)** and **(C)**. Data were presented as the mean ± standard deviation. *P < 0.05 and **P < 0.01 vs. the sham group;^#^P < 0.05 and ^##^P < 0.01vs. CLP group,n = 8.

The activation of ERK1/2 and NF‑κB is critical for the production of inflammatory cytokines such as TNF-α and IL-6, during endotoxemia or sepsis [16]. To measure the effect of dexmedetomidine on the activation of ERK1/2 and NF‑κB, lung tissues in each group were homogenized and the supernatant was collected. Compared with the sham group, CLP-induced sepsis dramatically promoted the activation of ERK1/2 and NF‑κB in CLP and yohimbine groups (P < 0.01). Dexmedetomidine with a dose of either 5 μg/kg or 10 μg/kg significantly inhibited CLP-induced ERK1/2 and NF‑κB activation and this effect could not be reversed by yohimbine(P < 0.01) (Figure 3).

## Discussion

Our study presented a new evidence that yohimbine, α_2_-adrenoceptor antagonists, couldn’t reverse the anti-inflammatory effect of dexmedetomidinein septic rats. Dexmedetomidine treatment (either 5 μg/kg or 10 μg/kg) could effectively decrease the production of IL-6 and TNF-α in the plasma and BALF of CLP-induced septic rats. Meanwhile, dexmedetomidine also inhibited the expression of TLR4 and MyD88 and the activation of ERK1/2 and NF-κB in the lung tissues of CLP-induced septic rats, indicating that the anti-inflammation effect of dexmedetomidine in sepsis maybe base on TLR4/MyD88/ERK1/2/NF-κB signal pathway.

Since dexmedetomidine is a selective α2-adrenergic receptor agonist, we wondered whether the anti-inflammation effect of dexmedetomidine depends on its α2-adrenoceptor activation effect, however so far there is few study focusing on this area. So we built yohimibine group to test our hypothesis, however, yohimbine failed to reverse the anti-inflammation effect of dexmedetomidine, indicating that the protective effect of dexmedetomidine on CLP-induced septic rats is independent of α_2_-adrenoceptor. So far, human trials and animal experiments have demonstrated that the generation of inflammatory cytokines, including TNF-α and IL-6, is one of the important characteristics of sepsis, and excessive inflammatory response can lead to acute lung injury [18]. As we know, dexmedetomidine is a potent and highly specific α_2_-adrenergic agonist with strong anti-inflammatory effects [19-22]. Qiao et al. found that dexmedetomidinecould lessen systemic inflammation and increase survival rate in septicor endotoxin-induced shock of rats [23,24]. Moreover, Venn et al. found that dexmedetomidine sedation could decrease the production of inflammatory cytokines, such as IL-1β,TNF-α and IL-6 in severe sepsis patients [25,26]. In our study, dexmedetomidine treatment at a dose of either 5 μg/kg or 10 μg/kg effectively reduced the generation of TNF-α and IL-6,and this effect,however, could not be reversed by yohimbine. These results showed the anti-inflammatory effect of dexmedetomidine in sepsis is independent of its α2-adrenoceptor activation ability.

TLR4 is a transmembrane receptor protein with extracellular leucine-rich repeated domains and a cytoplasmic signaling domain, specifically recognizing endogenous molecules released from damaged or ischemic tissues termed danger-associated molecular patterns (DAMPs), and pathogen associated molecular patterns(PAMPs), including sepsis [27,28]. TLR4 involves in immune responses, especially in the activation of innate immunity against foreign pathogens and microorganisms, in addition, TLR4 also triggers adaptative immunity [29-31]. Some studies have suggested that the association of TLR4 with myeloid differentiation factor 88(MyD88) may induce the production of IL-6 and TNF-α, which triggers inflammatory cascade reactions [32,33].

NF-κB is an important nuclear transcription factor, consisting of p50 and p65 subunits. It plays an important role in immune and inflammatory responses through regulating the expression of inflammatory cytokines, chemokines and adhesion molecules [34]. TLR/MyD88 signaling pathway activates mitogen-activated protein kinase (MAPK) family and NF-κB pathways, ultimately leading to the production of inflammatory mediators. Since the generationof IL-6 and TNF-α is highly relevant to LPS/TLR4/MyD88/MAPK/NF-κB signal pathway in sepsis, therefore, we verified the effect of dexmedetomidine on these signal molecules in CLP-induced septic rats. Dexmedetomidine at a concentration of either5 or 10 μg/kg could inhibit the expression of TLR4 and MyD88, and the activation of ERK1/2 and NF-κB in CLP-induced sepsis.

## Conclusions

In summary, this study demonstrated that CLP-induced sepsis promoted the expression of TLR4 and Myd88 and the activation of ERK1/2 and NF-κB in lung tissue, and ultimately induced the generation of TNF-α and IL-6 in BALF and plasma. However, these changes of CLP-induced sepsis could be partially inhibited by dexmedetomidine treatment and this inhibitory of dexmedetomidine could not be reversed by yohimbine. All these results indicated that dexmedetomidine treatment can effectively reduce the generation of inflammatory mediators in the plasma and BALF of CLP-induced septic rats. This effect of dexmedetomidine is independent of α_2_-adrenoceptor and probably is related to TLR4/MyD88/MAPK/ NF-κB signaling pathway.
